# Unmeasured improvement work: the lack of routinely collected, service-related data in NHS endoscopy units in England involved in "modernisation"

**DOI:** 10.1186/1472-6963-8-20

**Published:** 2008-01-24

**Authors:** Kymberley Thorne, Hayley A Hutchings, Glyn Elwyn

**Affiliations:** 1 School of Medicine, Swansea University, Swansea, UK; 2 Department of Primary Care and Public Health, Cardiff University, Cardiff, UK

## Abstract

**Background:**

The availability of routinely collected service-related endoscopy data from NHS endoscopy units has never been quantified.

**Methods:**

This retrospective observational study asked 19 endoscopy units to submit copies of all in-house, service-related endoscopy data that had been routinely collected by the unit – *Referral numbers, Activity, Number of patients waiting *and *Number of lost slots*. Nine of the endoscopy units had previously participated in the Modernising Endoscopy Services (MES) project during 2003 to redesign their endoscopy services. These MES sites had access to additional funding and data collection software. The other ten (Control sites) had modernised independently. All data was requested in two phases and corresponded to eight specific time points between January 2003 and April 2006.

**Results:**

Only eight of 19 endoscopy units submitted routinely collected, service-related data. Another site's data was collected specifically for the study. A further two units claimed to routinely collect service-related data but did not submit any to the study. The remaining eight did not collect any service-related endoscopy data routinely and liaised with their Trust for data. Of the eight sites submitting service-related data, only three were MES project sites. Of these three, the data variables collected were limited and none collected the complete set of endoscopy data variables requested. Of the other five sites, two collected all four endoscopy data types. Data for the three MES project sites went back as far as January 2003, whilst the five Control sites were only able to submit data from December 2003 onwards.

**Conclusion:**

There was a lack of service-related endoscopy data routinely collected by the study sites, especially those who had participated in the MES project. Without this data, NHS endoscopy services cannot have a true understanding of their services, cannot identify problems and cannot measure the impact of any changes. With the increasing pressures placed on NHS endoscopy services, the need to effectively inform redesign plans is paramount. We recommend the compulsory collection of service-related endoscopy data by all NHS endoscopy units using a standardised format with rigorous guidelines.

## Background

Good quality, routinely collected data are essential for the analysis of a process in order to understand and improve it, and to reduce the effect of "management by opinion" [1]. Unfortunately, the NHS does not have a good track history for collecting accurate, analysable service-related data on a routine basis [2] due to a historical lack in investment and interest [3]. This has made service evaluation and evidence-based policy development extremely difficult.

Most independent studies evaluating NHS endoscopy services using routine NHS endoscopy data corresponding to English NHS Trusts rely on patient-level data provided by independent organisations such as the Department of Health (DoH) and Hospital Episode Statistics (HES). However, there are many reasons why these warehouses should not be considered to be holding the "gold standard" of datasets. Firstly, these datasets are amassed from data submitted by NHS Trusts to the NHS-Wide Clearing Service in a standardised format and whilst they are sufficient to enable reasonably accurate judgements on NHS performance based on data trends, they are often not collected in categories suitable for in-house or independent analyses (e.g. procedure types, referral types). Secondly, it is not compulsory to split data down any further than Trust level, making the evaluation of endoscopy services in a single hospital within a Trust impossible where the Trust has more than one endoscopy unit. Thirdly, endoscopies performed in outpatient clinics were not captured by HES until 2003 and even then, many procedures were not coded for, which means that activity may be under-represented in the HES dataset. Finally, there have been documented cases of large error rates in the accuracy [4-7] and the reproducibility [8] of NHS Trust datasets in a variety of medical fields, which casts serious doubts on the validity of Trust datasets, especially when evidence of the deliberate falsification of records has also been reported [9].

Based on these reasons, there is a real need for NHS endoscopy units to introduce unit-level data collection for analysis by both themselves and independent researchers in order to better understand and improve their own working practices, to produce "stronger" evidence-based policy changes and to provide accurate figures for national and local audits. However, it is not clear whether NHS endoscopy services routinely collect service-related data in terms of monthly counts for demand, activity, waiting lists and lost appointment slots, since it is not a compulsory facet of the service. There has been no peer-reviewed literature published to date that makes use of routinely collected, service-related endoscopy data at individual hospital level, which leads us to the question, is this type of data being routinely collected by NHS endoscopy units in England?

The NHS Modernisation Agency initiated the Modernising Endoscopy Service (MES) project which ran from January to December 2003 involving 26 NHS endoscopy units in England. These sites were given £30,000 and data collection software called the MES Toolkit to facilitate the analysis and redesign of their endoscopy services [10]. The MES project advocated the need for regular, high quality data collection and process mapping to better understand endoscopy services and identify the true cause of any problems in order to target redesign plans effectively. To achieve this, the project enforced a rigorous data collection regime on all study sites, encompassing demand, activity, waiting lists, capacity and lost slots [11]. Data was uploaded to the project base on a monthly basis for remote analysis, although the data was available for the sites to analyse themselves if they so wished.

An independent research study funded by the National Institute for Health Research Service Delivery and Organisation Programme (formally the NHS Service Delivery and Organisation) is currently evaluating the effect of the MES project – the **E**valuating I**N**novations **I**n **G**astroenterology by the NHS **M**odernisation **A**gency (ENIGMA) study (SDO 46/2003). It compared a random selection of 10 endoscopy units that participated in the MES project (Intervention sites) with 10 randomly selected English NHS endoscopy units that modernised independently (Control sites). One aspect of the evaluation involved the collection and analysis of service-related endoscopy data encompassing demand, activity, waiting lists and lost slots from all study sites at eight separate time points between January 2003 and April 2006. The purpose of this paper is to describe the availability of the service-related data that had been routinely collected by each ENIGMA study site and to describe the types of data submitted.

## Methods

Requests for copies of routinely collected, service-related endoscopy data were made to the ENIGMA contact based in the endoscopy units of the nine Intervention sites (one had been withdrawn from ENIGMA due to problems with patient recruitment) and 10 Control sites participating in the ENIGMA study. The study had previously selected these 20 sites at random by ranking by bed number and selecting using interval choice using a randomly assigned number. There was no prior knowledge regarding their routine endoscopy data collection practices.

The data request for this study specified routinely collected data to prevent specific data retrieval from the Patient Administration System (PAS). It was designed to test the availability of routinely collected, service-related data in each unit to determine which units were in the position to immediately analyse and evaluate their services.

The request for service-related data encompassed counts for *Referral numbers, Activity, Number of patients waiting *and *Number of lost slots*. The data request was done retrospectively in two phases. The first phase collected data pertaining to January, June and December 2003, April and November 2004, and April 2005, whilst the second phase collected data pertaining to October 2005 and April 2006.

All data submitted to the study was examined by the researcher to check that it had been routinely collected by the endoscopy unit and whether any had been compiled specifically for the purpose of this study. The evaluation was based on visual examination of data files using the creation date, format and content of the electronic files as a guide, followed by verbal verification via direct communication with the person responsible for sending the data from each study site.

Data was split according to whether it was submitted by an Intervention or Control site. Data types submitted were counted to identify which variables were routinely collected out of the four types requested, and which were not. The researcher also recorded which of the time points requested were completed by each site.

## Results

**Figure 1:**
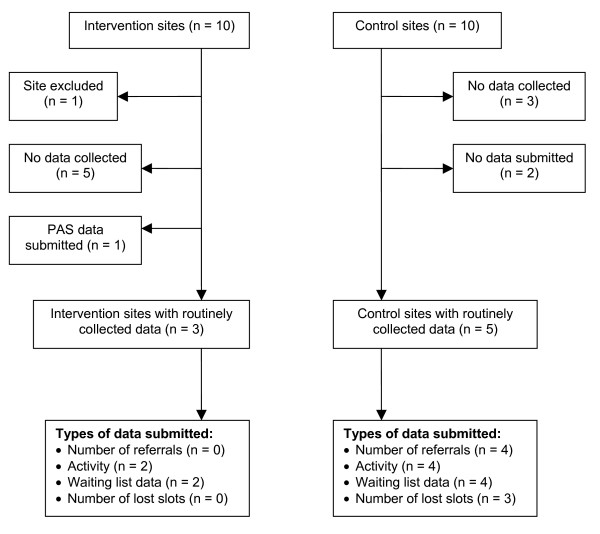
Flowchart describing the data availability in Intervention and Control sites.

All 19 endoscopy units were successfully contacted by the researcher by telephone or Email and the results are illustrated in Figure 1. Nine endoscopy units submitted at least one type of service-related data, but only eight datasets had been routinely collected by the endoscopy units. The remaining site verbally confirmed that they had deliberately compiled a file relating to the data request using their PAS because the dataset was not routinely collected within the unit. Of the remaining 10 sites, two commented that data was routinely collected within the department but it was never submitted and of the other eight sites, none routinely collected any endoscopy service-related data and relied upon their Trust Information Department to provide service-related data on request. Of the eight sites submitting routinely collected service-related data, only three were Intervention sites, whilst the other five were Control sites. The two sites who did not submit their routine data were both Control sites.

Table 1 shows a breakdown of the types of routinely collected service-related data submitted by both Intervention and Control sites. Only two sites collected all four data types, both of which were Controls. A further three sites collected only one data type (two Intervention sites and one Control site). This was *Activity *for one site and *Number of patients waiting *for the other two sites. Of the three Intervention sites with data, two collected *Activity *data and two collected *Number of patients waiting *but none collected *Referral numbers *or *Number of lost slots*. Of the five Control sites with data, four collected *Referral numbers, Activity *and *Number of patients waiting*, whilst three collected *Number of lost slots*.

**Table 1:** Breakdown of the type of endoscopy data collected by each of the eight study sites submitting routinely collected data and the earliest time point with data from that site.

**Site type**	**No. referrals**	**Activity**	**No. patients waiting**	**No. lost slots**	**Earliest time period with data**
Intervention	No	Yes	Yes	No	Jan-03
	No	No	Yes	No	Jan-03
	No	Yes	No	No	Jan-03
Control	Yes	Yes	Yes	Yes	Dec-03
	Yes	Yes	Yes	Yes	Dec-03
	Yes	Yes	No	Yes	Apr-04
	Yes	Yes	Yes	No	Dec-03
	No	No	Yes	No	Apr-04

All three Intervention sites were able to submit data as far back as January 2003, whilst the five Control sites had started routine data collection prior to either December 2003 or April 2004. In all eight cases the data submitted was complete as far as the last time point requested, April 2006.

Most data submitted was split according to procedure types and was often further split within the data type (e.g. types of referrals, etc), and was collected per calendar month, making a more detailed analysis of services possible for each site if necessary. In all cases the data was collated within Excel spreadsheets.

## Discussion

During the course of the study, we found that the routine collection of service-related endoscopy data in a selection of NHS endoscopy units in England was limited, with only eight of 19 units collecting at least one data type. Where collected, it was highly varied in its content, although there was a good level of detail in the data to allow a more focussed analysis of services.

This study is unique in its quantification of the availability of service-related data that had been routinely collected within English NHS endoscopy units. We were only able to identify four published studies using Pubmed that analysed service-related endoscopy data but in all cases, the data had been compiled specifically for the studies and were not routinely collected datasets [12-15]. Whilst it is entirely possible that someone in each unit held routine data that was not submitted to this study due to our external position, every effort was made to obtain data from these units and the researcher was confident that all potential avenues of routinely collected, service-related data were explored thoroughly.

Six of the nine Intervention sites did not routinely collect service-related data either during or after the MES project. This was surprising, since the MES project based its redesign theories on the collection and analysis of accurate, measurable service-related data prior to the implementation of targeted innovations to improve services from a patient-centred perspective to address targets published by the NHS Cancer Plan [16]. Informal discussions with Intervention site endoscopy staff found that completion of the MES Toolkit during 2003 was done by retrieving data using PAS or by liaising with the Trust. Unfortunately, the value of the data collected for the MES project did not seem to have motivated the staff into routinely collecting similar datasets following its close. This may have been because the MES Toolkit data was analysed remotely by the MES project team. NHS staff are often not trained to collect and analyse data to evaluate their services and so, may not have identified its true potential for identifying problem areas for effective, targeted changes or for measuring the impact of a modernisation strategy on services as a whole. Alternatively, they may have wished to continue collecting data but had no confidence in its accurate analysis. Also, the rigorous nature of the data collection required for the MES Toolkit may have deterred staff from continuing with such a time-consuming, complicated piece of software. Even so, these sites could have taken on board the messages of the MES project and embarked upon their own in-house data collection regime. Unfortunately, any further exploration of the reasons for the lack of routinely collected data in these sites was beyond the original scope of this study.

Another surprising aspect of this study was the degree to which Control sites had initiated their own, in-house data collection protocols compared to the Intervention sites. The Control sites were all aware of the MES project because they had originally applied to take part but had been rejected. It is possible that the messages of accurate data collection advocated by the MES project were disseminated to these sites and they began their own, less rigorous data collection processes, albeit later in the study. The author was able to confirm that these sites had not collected data for the Webtool [17], a freely available web-based version of the MES Toolkit, meaning that all data collection was designed and implemented in-house.

During the course of the ENIGMA study, we were concerned to hear from some Trust personnel about the degree of potential coding ambiguities in their own Trust-held endoscopy datasets, although there was no published evidence available to support this. Since the Trust-held datasets are collated from PAS data, we would question whether these datasets were accurate in the eight sites that relied on Trust-held data or their PAS interrogations.

The need for good quality routine data in the NHS has been widely acknowledged based on independent assessment of current data collection practices [1,7,18]. However, there is currently no national or local impetus to routinely collect data for NHS endoscopy staff, even in light of the MES project, which reported significant improvements to MES site services attributed partly to high quality data collection and analysis that was used to inform redesign plans [10]. It may be that data and data quality is too often seen as a function only of the IT department [18]. Alternatively, if there is little understanding of how to collect and analyse the data, NHS staff may not be motivated enough to bear the additional workload involved. Unfortunately, only a qualitative follow-up can comprehensively provide the real reasons for the data deficit identified in these sites.

With increasing demands on NHS endoscopy services from Two Week Rule (TWR) cancer referrals and the Bowel Cancer Screening programme, we would question how the service can maintain a basic standard, let alone become more efficient, if it does not understand how it works and where underlying problems exist in order to target redesign plans effectively. Even the most basic understanding of the demand and activity within the endoscopy unit can identify underused resources or potential problems for further investigation, as well as providing a baseline measurement with which to measure the impact of a change to the service. It can also provide an invaluable source of evidence when submitting bids for funding, all of which can make the effort of establishing a data collection regime worthwhile.

If this situation arose within the industrial sector whereby no routine measurements of a process were made, the company would fail. Perhaps the lack of business experience in some NHS managers can go some way towards explaining the ineffective working practices of many NHS services, not just endoscopy, as many NHS managers are not trained in industry-based redesign concepts such as Business Process Reengineering [19], Total Quality Management [20], Lean thinking [21,22] and the Theory of Constraints [23,24], all of which advocate data collection and analysis as the basis for improving a process/service.

The Audit Commission have recently published a report aimed at public services to improve the quality of their data [25]. If this could be used as a framework for the NHS to initiate an improved data collection strategy, perhaps the quality of NHS services would improve in line with its datasets? However, the author is keen to acknowledge that high quality data collection will not solve all the services' problems; it will only provide the tools to understand them. It can provide a platform from which managers can engage with other NHS staff to promote redesign initiatives and is invaluable in measuring the impact of any reforms to provide evidence of success to further motivate staff.

## Conclusion

We conclude that there was insufficient service-related endoscopy data being routinely collected by a selection of NHS endoscopy units in England. Furthermore, participation in the MES project did not seem to encourage the collection of data in MES sites following the project's close.

Based on the datasets received, it was clear that good quality, analysable data could be collected without great cost or time being invested once a system was in place. With this in mind, we would recommend the compulsory collection of basic endoscopy data by all NHS endoscopy units to facilitate the evaluation and improvement of services in line with other redesign strategies. It is vital that the data should be collected in line with rigorous guidelines and standardised definitions to ensure comparability and compatibility between units to ensure any nationwide evaluations are accurate. It is also important that each endoscopy unit has a member of staff who is responsible for routine data collection and is suitably trained in its analysis in order for the unit to reap the potential benefits.

All discussions concerning issues of data collection, or lack of, with the study sites were informal and no qualitative analysis was done. However, since this emerged as an important issue in this study, follow-up interviews with endoscopy staff would be the next step to understanding why data are not routinely collected.

## Competing interests

The author(s) declare that they have no competing interests.

## Authors' contributions

KT conceived of the study, performed the literature review and drafted the manuscript. HAH and GE contributed to the design of the study and the literature review and were involved in drafting the manuscript. All authors read and approved the final manuscript.

## Pre-publication history

The pre-publication history for this paper can be accessed here:


